# The role of perceived minority-group status in the conspiracy beliefs of factual majority groups

**DOI:** 10.1098/rsos.221036

**Published:** 2023-10-18

**Authors:** Aleksander B. Gundersen, Sander van der Linden, Michał Piksa, Mikołaj Morzy, Jan Piasecki, Rafał Ryguła, Paweł Gwiaździński, Karolina Noworyta, Jonas R. Kunst

**Affiliations:** ^1^ Department of Psychology, University of Oslo, Postboks 1094 Blindern, 0317 Oslo, Norway; ^2^ Department of Psychology, University of Cambridge, Cambridge, Cambridgeshire, UK; ^3^ Department of Pharmacology, Maj Institute of Pharmacology Polish Academy of Sciences, Krakow, Poland; ^4^ Institute of Computing Science, Poznan University of Technology, Poznan, Poland; ^5^ Department of Philosophy and Bioethics, Jagiellonian University Medical College, Kraków, Poland; ^6^ Department of Philosophy and Sociology, Pedagogical University of Krakow, Kraków, Poland

**Keywords:** conspiracy beliefs, COVID-19, gender, minority groups, misinformation

## Abstract

Research suggests that minority-group members sometimes are more susceptible to misinformation. Two complementary studies examined the influence of perceived minority status on susceptibility to misinformation and conspiracy beliefs. In study 1 (*n* = 2140), the perception of belonging to a minority group, rather than factually belonging to it, was most consistently related with an increased susceptibility to COVID-19 misinformation across national samples from the USA, the UK, Germany and Poland. Specifically, perceiving that one belongs to a gender minority group particularly predicted susceptibility to misinformation when participants factually did not belong to it. In pre-registered study 2 (*n* = 1823), an experiment aiming to manipulate the minority perceptions of men failed to influence conspiracy beliefs in the predicted direction. However, pre-registered correlational analyses showed that men who view themselves as a gender minority were more prone to gender conspiracy beliefs and exhibited a heightened conspiracy mentality. This effect was correlationally mediated by increased feelings of system identity threat, collective narcissism, group relative deprivation and actively open-minded thinking. Especially, the perception of being a minority in terms of power and influence (as compared to numerically) was linked to these outcomes. We discuss limitations and practical implications for countering misinformation.

## 1. The role of perceived minority-group status in the conspiracy beliefs of factual majority groups

Misinformation and conspiracy theories have received increasing attention from researchers, journalists, policymakers and the wider public in recent years. This rise in interest is partly based on the viewpoint that misinformation and conspiracy theories can have serious negative individual and societal outcomes that must be mitigated. For example, conspiracy beliefs have been linked to the resurgence of vaccine-preventable diseases [1], anti-science attitudes [2] and promotion of prejudice [3]. Therefore, research into how misinformation and conspiracy theories spread [4,5], determinants of believing in them [6–8] and how to counter them [9,10] have proliferated in recent years. However, despite the rapid growth of the field, it still remains in its infancy [11].

Psychological research on people's susceptibility to believe in misinformation and conspiracy beliefs has highlighted the role of various individual and contextual factors. For instance, several cognitive factors (e.g. intuitive thinking, cognitive failures) and socio-affective factors (e.g. source cues, emotion, worldview) have been put forward as some of the main drivers of false beliefs (for a review, see [6]). Similarly, various individual, intergroup and national-level influences have been linked to conspiracy beliefs [12]. Moreover, previous work has also pointed to demographic factors related to increased misinformation susceptibility such as having a minority background [13]. Here, it has been found that ethnic and religious minorities hold stronger conspiracy beliefs [14], and susceptibility to health misinformation has been associated with religiosity, having a conservative political ideology, and being part of a racial minority [15]. Individuals with low socio-economic status have also been found to hold more coronavirus conspiracy beliefs [16].

While suggestive, existing research faces some critical limitations. First and foremost, it does not differentiate between *perceiving* oneself to be part of a minority group versus *factually* belonging to it. The role of minority perceptions was demonstrated in a study by Roozenbeek *et al*. [17]. Here, high self-perceived minority status was significantly associated with an increased susceptibility to COVID-19 misinformation. However, the question assessing self-perceived minority status was framed generically without reference to a specific group. Thus, it could include *any* self-identified minority group, limiting the interpretability of these results. For example, one could score high on this scale whether one identified as part of an ethnic, religious, gender or political minority group. Moreover, the researchers did not compare the effects and potential interactions between perceived and factual minority status.

It is important to note that we in the present manuscript define minority perceptions as the cognitive evaluation that one's group is a minority in society. In the second study, we further distinguish between perceiving oneself as a minority in terms of numbers or in terms of power. Although these perceptions are conceptually related to constructs such as collective victimhood [18] or relative deprivation [19], they differ in that we conceptualize minority perceptions as not pre-requiring a certain set of negative affective responses. For instance, a person who perceives their own group as a minority does not need to have a negative reaction towards this minority status (e.g. some men may perceive a minority of men in traditionally male-dominated domains as something positive). By contrast, a negative affective reaction is essential to and directly assessed by constructs such as relative deprivation or collective victimhood. However, acknowledging the close conceptual relationship between the variables, we test whether variables such as relative deprivation mediate the effects of minority perceptions in study 2. Indeed, perceptions of belonging to a minority group can be rooted in feelings of powerlessness, marginalization and disadvantage [20–22]. In general, perceptions of such deprived life circumstances have been associated with an increased susceptibility to misinformation (see [23] for a review). Feelings of powerlessness have been associated with believing in conspiracy theories [24,25], and feelings of powerlessness have also been found to mediate the relationship between low education and conspiracy beliefs [26]. Relatedly, perceptions of prejudice and discrimination have been found to mediate the relationship between belonging to a marginalized minority group and believing in conspiracy theories [27]. Furthermore, feelings of personal and group-based deprivation have been found to contribute to conspiracy beliefs among ethnic and religious minorities [14].

However, the feeling of belonging to a minority group does not always correspond to factual group memberships. A growing body of research has found that White Americans fear a ‘majority–minority’ shift that threatens their dominant social, economic, political and cultural status (see [28] for a review). Relatedly, a survey showed that 55% of White Americans believed there is discrimination against White people [29]. Taken together, these findings may indicate that some White people perceive themselves to be part of a minority group. Similarly, members of the Christian majority in the USA have been found to perceive themselves as a minority [20]. Research shows that often an individual's perception of their group's position relative to other groups, rather than its actual position and size, predicts psychological outcomes. For instance, it has been suggested that well-being is perceived mainly in relative terms rather than in absolute terms [30], that relative status predicts more hostility than absolute status [31], and that religious majority members who perceive themselves as a minority report more stress via heightened perceptions of discrimination than religious majority members who do not perceive themselves as part of a minority group [20]. Thus, it is possible that the perception of belonging to a minority group may be more predictive of a susceptibility to misinformation than factually belonging to it.

## 2. Overview of the current studies

With this background, the present research aimed to address two gaps in the literature. First, and most critically, the extent to which having a *self-perceived* minority status may have different associations with conspiracy tendencies than *factually* belonging to a minority group has not been tested. Second, whether these effects depend on the type of group in question remains unknown. To fill these gaps, study 1 tested how peoples' self-perceived and factual minority statuses based on a broad range of group dimensions (i.e. ethnicity, gender, sexual, religious, political, social class and region of residence) predict susceptibility to COVID-19 misinformation. To investigate whether these potential effects are dependent on the national context or can be generalized across (Western) nations, we conducted this research with participants from the USA, the UK, Poland and Germany. These countries differ in quality of democracy [32], freedom of the press [33], government response stringency [34], COVID-19 vaccine uptake [35] and trust in institutions [36]. Thus, by investigating to what extent effects are similar or different among the four countries, we obtain important information about the generalizability of effects.

Next, in a pre-registered experiment with the USA sample (study 2), we aimed to expand on the results from study 1 by testing whether self-perceived minority status can be experimentally manipulated and is causally related to misinformation susceptibility. Furthermore, we wanted to test whether this effect is moderated by political orientation and preference for social hierarchies (i.e. social dominance orientation (SDO); [37]). Previous findings have shown that perceived ingroup disadvantage [38–40] and actively open-minded thinking (AOT) [41] are linked to susceptibility to misinformation. Thus, we also tested whether the effect of perceived minority status on misinformation susceptibility was mediated by system identity threat [42], collective narcissism [43], group relative deprivation [19] and AOT [44].

## 3. Study 1

### 3.1. Method

#### 3.1.1. Participants

Between 16 June and 9 August 2021, we recruited participants from the USA (*n* = 492), the UK (*n* = 600), Poland (*n* = 558) and Germany (*n* = 490). All samples were obtained through the International Organization for Standardization-certified online survey platform Respondi (for the UK and Germany) and CloudResearch (for the USA and Poland). Participants were quota sampled based on gender, age, income and education, resulting in samples that were close to representative in terms of most core demographic variables (table 1). A total of 16.8% of the participants were excluded as they failed two attention checks (see table 2 for a description).

**Table 1.** Descriptive statistics of participants by country in study 1.

	USA	UK	Poland	Germany	total
*n*	492	600	558	490	2140
male (%)	242 (49.2)	298 (49.7)	270 (48.4)	243 (49.6)	1053 (49.2)
female (%)	247 (50.2)	301 (50.2)	287 (51.4)	245 (50.0)	1080 (50.5)
other (%)	3 (0.6)	1 (0.2)	1 (0.2)	2 (0.4)	7 (0.3)
age, *M* (s.d.)	46.56 (18.39)	50.44 (16.84)	44.50 (17.06)	47.25 (17.70)	47.28 (17.59)
18–24	74 (15.0)	44 (7.3)	67 (12.0)	60 (12.2)	245 (11.4)
25–54	239 (48.6)	308 (51.3)	290 (52.0)	226 (46.1)	1063 (49.7)
55–64	78 (15.9)	106 (17.7)	91 (16.3)	91 (18.6)	366 (17.1)
65 < (%)	101 (20.5)	142 (23.7)	110 (19.7)	113 (23.1)	466 (21.8)
education					
below upper secondary (%)	35 (7.1)	126 (21.0)	13 (2.3)	64 (13.1)	238 (11.1)
upper secondary (%)	214 (43.5)	149 (24.8)	353 (63.3)	279 (57.0)	994 (46.5)
tertiary (%)	243 (49.4)	325 (54.2)	192 (34.4)	147 (29.9)	906 (42.4)
income relative to median					
low (%)	174 (35.4)	107 (17.8)	183 (32.8)	95 (19.4)	559 (26.1)
middle (%)	163 (33.1)	412 (68.7)	316 (56.6)	313 (63.9)	1204 (56.3)
high (%)	155 (31.5)	81 (13.5)	59 (10.6)	82 (16.7)	377 (17.6)
region of residence					
predominantly urban	231 (47.0)	277 (46.2)	449 (80.8)	273 (55.7)	1230 (57.5)
predominantly rural–suburb	190 (38.6)	224 (37.3)	61 (11.0)	160 (32.7)	635 (29.7)
predominantly rural–remote	71 (14.4)	99 (16.5)	46 (8.3)	57 (11.6)	293 (12.8)
religious affiliation					
non-affiliated	188 (38.5)	271 (45.4)	128 (23.1)	227 (46.4)	814 (38.3)
Roman Catholic	97 (19.9)	68 (11.4)	385 (69.6)	108 (22.1)	658 (30.9)
Orthodox	3 (0.6)	5 (0.8)	10 (1.8)	4 (0.8)	22 (1.0)
Jewish	13 (2.7)	5 (0.8)	1 (0.2)	2 (0.4)	21 (1.0)
Muslim	7 (1.4)	19 (3.2)	2 (0.4)	9 (1.8)	37 (1.7)
Hindu		6 (1.0)		1 (0.2)	7 (0.3)
Buddhist	4 (0.8)	2 (0.3)	1 (0.2)	2 (0.4)	9 (0.4)
Protestant	107 (21.9)	172 (28.8)	9 (1.6)	125 (25.6)	413 (6.9)
other	69 (14.1)	49 (8.2)	17 (3.1)	11 (2.2)	146 (6.9)
employment status					
employed full time	160 (32.5)	289 (48.2)	310 (55.6)	230 (46.9)	989 (46.2)
employed part time	56 (11.4)	64 (10.7)	45 (8.1)	56 (11.4)	211 (10.3)
unemployed, looking for work	55 (11.2)	13 (2.2)	25 (4.5)	7 (1.4)	100 (4.7)
unemployed, not looking for work	37 (7.5)	34 (5.7)	15 (2.7)	17 (3.5)	103 (4.8)
retired	112 (22.8)	150 (25.0)	128 (22.9)	120 (24.5)	510 (23.8)
student	25 (5.1)	27 (4.5)	28 (5.0)	54 (11.0)	134 (6.3)
disabled	47 (9.6)	23 (3.8)	7 (1.3)	6 (1.2)	83 (3.9)
ethnicity, *n* (%)					
majority members	388 (79.0)	524 (87.3)	524 (93.9)	446 (91.2)	1882 (88.0)
minority members	103 (21.0)^a^	76 (12.7)^b^	34 (6.1)^c^	43 (8.8)^d^	256 (12.0)

^a^Black/African American (*n* = 44/9.0%), Asian (*n* = 19/3.9%), American Indian or Alaska native (*n* = 2/0.4%), Hispanic (*n* = 28/5.7%), other (*n* = 3/0.3%).

^b^Black/African/Caribbean/black British (*n* = 15/2.5%), Asian/Asian British: Indian (*n* = 14/2.3%), Asian/Asian British: Pakistani (*n* = 11/1.8%), mixed (*n* = 13/2.2%), other (*n* = 23/3.8%).

^c^Silesian (*n* = 20/3.6%), German (*n* = 3/0.5%), Ukrainian (*n* = 4/0.7%), other (*n* = 7/1.3%).

^d^Urkish (*n* = 5/1.0%), Polish (*n* = 6/1.2%), other (*n* = 32/6.5%).

**Table 2.** Description of the main study variables in study 1. Note: each factual minority status coded as 0—majority, 1—minority. The distribution of perceived minority status as a function of factual minority status across countries is displayed in the electronic supplementary material, table S3.)

variable	example item	response format and categorization
susceptibility to misinformation^a^	‘the new 5G network may be making us more susceptible to the virus.’ (five items; USA: *α* = 0.89; UK: *α* = 0.87; Poland: *α* = 0.83; Germany: *α* = 0.80)	6-point Likert scale from 1 (*very unreliable*) to 6 (*very reliable*)
self-perceived minority status	‘to what extent do you consider yourself part of a minority group based on your… [*ethnicity, religion, political views, sexual orientation, gender identity, social class, region of living*]’ (seven single items in total)	7-point Likert scale from 1 (*strongly disagree*) to 7 (*strongly agree*)
factual minority status (each 1 item)		
ethnicity	‘please indicate your ethnic origin’	coded as 1 (*ethnic minority*) or 0 (*ethnic majority*)
religion/atheist	‘do you belong to a religious denomination? If yes, which one?’	two dummy variables^b^, one coded as 1 (*religious minority*) or 0 (*religious majority*). The other coded as 1 (*atheist/non-religious*) or 0 (*religious majority*)
political orientation	‘please indicate below to what extent you consider yourself as liberal or conservative in terms of political attitudes’	11-point Likert scale from 0 (*very left-wing/liberal*) to 10 (*very right-wing/conservative*). Two dummy variables^c^ coded as 1 (*political minority*) or 0 (*political majority*)
sexual orientation	‘what is your sexual orientation?’	coded as 1 (*non-heterosexuals*) or 0 (*heterosexuals*)
gender identity	‘what is your gender?’	coded as 1 (*women/non-binary/other*) or 0 (*men*)
social class^d^	‘what is your highest level of education?’ and ‘what is your annual income?’	coded as 1 (*low social class*) or 0 (*not low social class*)
region of residence	‘what best describes your area of living?’	coded as 1 (*rural residents*) or 0 (*urban residents*)
attention check^e^	‘it is important that you pay attention to the survey. Please select ‘somewhat agree’	two items interspersed within other measures on 5- and 7-point Likert scales

^a^Adopted from Roozenbeek *et al.* [17]. The susceptibility to misinformation scale was computed by averaging the six false statements (see the electronic supplementary material, table S1 for all items). One false statement was dropped to improve measurement invariance and achieve metric invariance (see the electronic supplementary material, S1 and table S2) for the remaining five-item scale.

^b^By simultaneously introducing both dummy variables to the model, we were able to test the effect of belonging to a religious minority group or being non-religious relative to belonging to the religious majority group.

^c^By entering both dummies into the models we compared the effect of belonging to the left-wing and the right-wing minority compared to the political midsection.

^d^Participants reporting an income of less than 66% of the median income who *also* had not completed high school were coded as the minority group.

^e^Participants who failed both attention checks were excluded from the final sample.

#### 3.1.2. Procedure and materials

This research was approved by the Institutional Review Board of the department of the first author (no. 12 660 124). The instruments described and analysed in the present study were part of a larger dataset on COVID-19 attitudes and preventive behaviour. All instruments were forward-back translated from English to Polish and German. Belief in misinformation and self-perceived minority status measures were presented in random order. The dataset and R code are available in the electronic supplementary material via the Open Science Framework (OSF): https://osf.io/65jrv/?view_only=4cc3c300fe77470b86cc20f1c098e1ae.

The instruments are presented in table 2. Participants were asked to indicate to what extent they perceived themselves as part of a minority group based on seven different dimensions. To determine whether they factually belonged to minority or majority groups on the same seven dimensions, we categorized them based on the demographic information they provided following the criteria in table 3.

**Table 3.** Criteria for and percentages of participants categorized into the different factual minority groups in study 1.

factual minority status	categorization
ethnicity (12.0%)	participants were categorized as either factually belonging to an ethnic minority group or the ethnic majority group based on an ordered listing of the ethnic majority and minority groups within each respective country [45]. See note accompanying table 1 for details
religious affiliation (11.3% religious minority/38.0% non-religious)	in the USA and Germany, Protestants and Catholics were considered the religious majority group [46]. For the UK, the religious majority are Christians generally (includes Anglican, Roman Catholic, Presbyterian and Methodist), while in Poland it is Catholics. Participants not belonging to any of these religious denominations in their country were thus coded as part of a factual religious minority group
political orientation (7.8% left-wing/7.3% right-wing)	in two separate dummy variables, participants with either a very left-wing/liberal political view (scoring 0 or 1 on the scale) or a very right-wing/conservative political view (scoring 9 or 10 on the scale) were categorized as a factual political minority and compared to the rest of the participants
sexual orientation (8.6%)	participants indicating another sexual orientation than heterosexual were categorized as part of a factual minority group and compared to heterosexuals
gender identity (50.6%)	although women are not numerically a minority group, one can argue that they constitute a minority in terms of power and status as they experience social inequalities relative to men in many societies including those considered in this research. Therefore, we coded participants who identified as women or other-gendered/non-binary as a minority group and men as the majority group. Please note that the number of non-binary responses (*n* = 7) did not allow us to compare it to binary responses
social class (5.4%)	a common way to categorize participants as belonging to the lower class in society is to operationalize low class as having a relative low income [47] and holding a low level of educational attainment (e.g. [48]). In line with this, participants were grouped into the minority group (i.e. low social class) or majority group (remaining participants) based on their income and education level relative to others in their country
region of residence (42.5%)	across the countries we sampled, the majority of the population live in urban areas (USA = 82.9%; UK = 84.2%; Poland = 60.1%; Germany = 77.5%; [49]). Hence, participants not living in an urban area were categorized as a part of a factual minority group based on region of residence

#### 3.1.3. Analyses

Given our multi-national sample and the relatively large number of predictors, a three-step hierarchical ANCOVA was conducted. In the first step (model 1), we tested for the main effects of self-perceived and factual minority variables. In the second step (model 2), we added interaction terms between the self-perceived and factual minority statuses for each minority dimension to test whether the effect of perceiving that one belongs to a minority depended on whether one factually belonged to it. Here, we also added interactions of perceived and factual minority-group membership with country to test to what extent the effects were the same across the different samples. In the last step (model 3), we added three-way interactions between the self-perceived minority statuses, their corresponding factual minority statuses, and country. We control for age and education in all analyses as they have previously shown associations with beliefs in false information (e.g. [17,50]).^1^ Given the large number of tests, we present Holm-corrected *p-*values for the regression analyses.

### 3.2. Results

Before running the ANCOVAs, it was important to test for multicollinearity as one may expect the factual and self-perceived minority statuses to be correlated. Table 4 displays bivariate correlations among all study variables in the full sample weighted by country. None of the matching factual and self-perceived minority statuses suggested multicollinearity. However, collinearity statistics in the regressions indicated that multicollinearity was a concern for the measure of self-perceived minority status based on sexual orientation (tolerance = 0.23, variance inflation factor (VIF) = 4.27) and gender identity (tolerance = 0.24, VIF = 4.17). Hence, the measure of self-perceived minority status based on sexual orientation was excluded from the main analyses (for results with this measure included, see the electronic supplementary material, S2). Please note that results with self-perceived minority status based on one's sexual orientation largely resembled the findings with participants' self-perceived minority status based on their gender.

**Table 4.** Bivariate correlations for study variables weighted by country in study 1.

variables	1	2	3	4	5	6	7	8	9	10	11	12	13	14	15	16	17	18
1. age	—																	
2. education	−0.05*	—																
factual minority status																		
3. ethnicity^a^	−0.21***	−0.02	—															
4. religion^a^	−0.07**	−0.07**	0.22***	—														
5. atheism^a^	−0.08***	0.01	−0.02	−0.28***	—													
6. political left^a^	−0.04	−0.01	0.03	0.00	0.09***	—												
7. political right^a^	0.06*	−0.00	−0.03	0.03	−0.12***	−0.08***	—											
8. gender identity^a^	−0.01	−0.05*	.07**	.06**	−0.03	.07**	−0.03	—										
9. sexual orientation^a^	−0.23***	−0.04*	0.10***	0.05*	0.09***	0.17***	−0.07**	0.04	—									
10. social class^a^	0.08***	−0.21***	−0.01	0.01	0.01	0.03	−0.01	0.06**	0.05*	—								
11. region of residence^a^	0.05*	−0.05*	0.04	0.04	0.02	−0.09***	0.04	0.02	0.00	0.06**	—							
self-perceived minority status																		
12. ethnicity	−0.17***	−0.01	0.42***	0.18***	−0.10***	0.04	0.07**	0.00	0.04*	−0.00	0.03	—						
13. religion	−0.13***	−0.04	0.16***	0.29***	−0.10***	0.06**	0.11***	0.00	0.04	−0.01	−0.01	0.57***	—					
14. political views	−0.15***	−0.04	0.12***	0.11***	−0.05*	0.08***	0.12***	−0.06**	0.07**	−0.01	0.00	0.48***	0.65***	—				
15. gender identity	−0.18***	−0.06**	0.14***	0.09***	−0.06**	0.04*	0.09***	−0.01	0.11***	0.02	0.02	0.62***	0.54***	0.51***	—			
16. sexual orientation	−0.22***	−0.05*	0.12***	0.09***	−0.03	0.11***	0.07**	−0.01	0.38***	0.03	0.02	0.55***	0.53***	0.52***	0.81***	—		
17. social class	−0.16***	−0.07***	0.17***	0.14***	−0.09***	0.043*	0.10***	−0.00	0.08***	0.05*	0.01	0.60***	0.58***	0.61***	0.68***	0.61***	—	
18. region of residence	−0.15***	−0.07**	0.20***	0.14***	−0.09***	0.01	0.10***	−0.02	0.05*	0.03	0.01	0.64***	0.58***	0.57***	0.69***	0.61***	0.78***	—
19. COVID-19 misinformation	−0.16***	0.00	0.09***	0.06**	−0.15***	−0.05*	0.20***	−0.05*	−0.04	−0.04	−0.07**	0.38***	0.32***	0.32***	0.43***	0.37***	0.38***	0.41***

^a^0 = factual majority member and 1 = factual minority member.

*p < 0.05; **p < 0.01; ***p < 0.001.

The model results are presented in table 5. Across all countries, participants rated COVID-19 misinformation as relatively unreliable (*M*_pooled_ = 1.94, s.d. = 1.06). However, based on pairwise comparisons with Holm-corrected significance levels, susceptibility to misinformation was significantly higher in the USA (*M* = 2.01, s.e. = 0.09) than the UK (Δ*M* = 0.19, 95% confidence interval (CI) [0.04, 0.35], s.e. = 0.06, *p* = 0.003) and Germany (Δ*M* = 0.33, 95% CI [0.15, 0.51], s.e. = 0.07, *p* < 0.001). Susceptibility to misinformation in Poland was also significantly higher (*M* = 2.10, s.e. = 0.09) than in the UK (Δ*M* = 0.28, 95% CI [0.12, 0.44], s.e. = 0.06, *p* < 0.001) and Germany (Δ*M* = 0.42, 95% CI [0.25, 0.59], s.e. = 0.06, *p* < 0.001). There was also a significantly higher misinformation susceptibility in the UK (*M* = 1.85, s.e. = 0.09) than in Germany (Δ*M* = 0.14, 95% CI [−0.02, 0.30], s.e. = 0.06, *p* = 0.050. However, this last comparison should be interpreted with caution as the CI contains zero. There was no significant difference between the USA and Poland (*p* = 0.155).

**Table 5.** Hierarchical ANCOVA with all main effects, two-way interactions and three-way interactions in study 1. (Note: degrees of freedom (d.f.) = 1 for all variables, except for education (d.f. = 5), country (d.f. = 3) and two-way interactions with country (d.f. = 3). *p_h_* = Holm-corrected significance levels. Main effects and interaction effects were corrected familywise. Significant estimates are presented in italics.)

variable	model 1			model 2			model 3		
	*F*	*p_h_*	$\eta_{p}^{2}$	*F*	*p_h_*	$\eta_{p}^{2}$	*F*	*p_h_*	$\eta_{p}^{2}$
(intercept)	*156.70*	*<0.001*		*50.13*	*<0.001*		*27.25*	*<0.001*	
age	*22.82*	*<0.001*	*0.04*	*17.60*	*0.001*	*0.04*	*15.45*	*0.001*	*0.04*
education	2.94	0.096	0.01	2.84	0.221	0.01	2.46	0.404	0.01
country	*16.53*	*<0.001*	*0.10*	*8.65*	*<0.001*	*0.10*	4.55	0.053	0.11
factual minority status									
ethnicity	0.21	>0.999	0.00	0.39	>0.999	0.00	3.44	0.764	0.00
atheism	*9.36*	*0.025*	*0.02*	0.14	>0.999	0.02	0.59	>0.999	0.02
religion	0.13	>0.999	0.00	1.63	>0.999	0.00	0.29	>0.999	0.00
political left	*8.28*	*0.037*	*0.01*	0.41	>0.999	0.01	0.28	>0.999	0.01
political right	*56.80*	*<0.001*	*0.04*	1.27	>0.999	0.04	1.43	>0.999	0.04
gender identity	2.42	0.837	0.00	0.29	>0.999	0.00	0.52	>0.999	0.00
sexual orientation	*14.43*	*0.002*	*0.01*	3.96	0.606	0.01	5.04	0.348	0.01
social class	0.06	>0.999	0.00	0.88	>0.999	0.00	0.72	>0.999	0.00
region of residence	*9.21*	*0.025*	*0.00*	0.85	>0.999	0.00	1.40	>0.999	0.01
self-perceived minority status									
ethnicity	*11.95*	*0.007*	*0.10*	1.22	>0.999	0.10	0.16	>0.999	0.11
religion	0.12	>0.999	0.01	4.27	0.546	0.01	1.56	>0.999	0.01
political views	1.79	>0.999	0.01	0.09	>0.999	0.01	0.95	>0.999	0.01
gender identity	*55.44*	*<0.001*	*0.05*	*50.65*	*<0.001*	*0.05*	*52.08*	*<0.001*	*0.05*
social class	0.02	>0.999	0.00	0.39	>0.999	0.00	0.36	>0.999	0.00
region of residence	*12.48*	*0.005*	*0.01*	3.53	0.726	0.01	8.68	0.052	0.01
two-way interactions (factual minority status × self-perceived minority status)									
ethnicity × ethnicity				0.48	>0.999	0.00	4.74	0.739	0.00
atheism × religion				0.04	>0.999	0.00	0.50	>0.999	0.00
religion × religion				6.66	0.208	0.00	1.21	>0.999	0.00
political left × political views				1.45	>0.999	0.00	4.92	0.694	0.00
political right × political views				3.27	>0.999	0.00	0.61	>0.999	0.00
gender identity × gender identity				*14.85*	*0.003*	*0.01*	*13.50*	*0.008*	*0.01*
social class × social class				0.55	>0.999	0.00	0.19	>0.999	0.00
region of residence × region of residence				5.22	0.426	0.00	*11.87*	*0.17*	*0.00*
two-way interactions (factual minority status × country)									
ethnicity × country				0.50	>0.999	0.00	2.83	0.897	0.00
atheism × country				1.79	>0.999	0.00	0.71	>0.999	0.00
religion × country				0.30	>0.999	0.00	0.88	>0.999	0.00
political left × country				0.55	>0.999	0.00	0.48	>0.999	0.00
political right × country				1.84	>0.999	0.00	0.37	>0.999	0.00
gender identity × country				3.84	0.205	0.01	1.55	>0.999	0.01
sexual orientation × country				0.50	>0.999	0.00	0.41	>0.999	0.00
social class × country				0.37	>0.999	0.00	0.63	>0.999	0.00
region of residence × country				1.06	>0.999	0.00	0.54	>0.999	0.00
two-way interactions (self-perceived minority status × country)									
ethnicity × country				3.42	0.333	0.00	4.91	0.061	0.00
religion × country				2.53	>0.999	0.01	0.79	>0.999	0.01
political views × country				0.60	>0.999	0.00	0.57	>0.999	0.00
gender identity × country				2.37	>0.999	0.01	3.77	0.290	0.01
social class × country				1.12	>0.999	0.00	0.55	>0.999	0.00
region of residence × country				0.61	>0.999	0.00	1.37	>0.999	0.00
three-way interactions (factual minority status × self-perceived minority status × country)									
ethnicity × ethnicity × country							3.27	0.555	0.00
atheism × religion × country							0.67	>0.999	0.00
religion × religion × country							0.70	>0.999	0.00
political left × political views × country							1.42	>0.999	0.00
political right × political views × country							2.48	>0.999	0.00
gender identity × gender identity × country							1.61	>0.999	0.00
social class × social class × country							0.64	>0.999	0.00
region of residence × region of residence × country							2.68	>0.999	0.00

**Figure 1.**
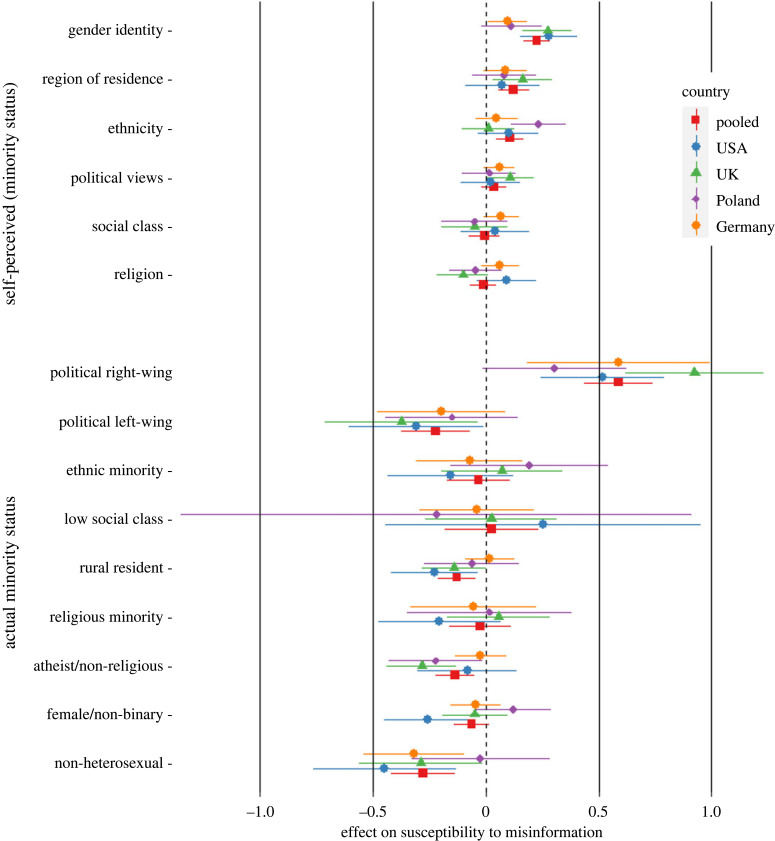
Effect of minority status on susceptibility to misinformation by country in study 1. Note: effects are standardized. Right of the dotted line indicates increased susceptibility to misinformation. Error bars represent 95% confidence intervals.

Figure 1 visualizes the results of the ordinary least-squares regression in the first step (model 1) of the hierarchical ANCOVA (table 5). This graph shows the effects across countries and for each country separately. Across countries, having a self-perceived minority status based on gender identity, region of residence and ethnicity were all significantly associated with an increased susceptibility to COVID-19 misinformation. By contrast, in terms of factual minority statuses, being non-heterosexual, non-religious, a rural resident or politically very left-wing were all significantly related to less susceptibility to misinformation. Of the factual minority statuses, only holding very right-wing political views was significantly associated with more susceptibility to misinformation. Following up on this effect, a correlation analysis showed that while holding very left-wing political views were unrelated or negatively associated with misinformation items, holding very right-wing political views was positively associated with all items (see the electronic supplementary material, table S6 for details). In terms of control variables, being older was associated with less susceptibility to misinformation, *β* = −0.10, 95% CI [−0.15, −0.06], *p* < 0.001, whereas the relationship between education level and misinformation susceptibility was non-significant.

#### 3.2.1. Two-way interactions

In the second step of the ANCOVA (table 5, model 2), we added two-way interactions to test whether (i) the effects of self-perceived and factual minority statuses differed between countries, and (ii) whether the effects of self-perceived and factual minority statuses for each type of minority group dimension were conditional on each other. We found no significant interactions between country and factual minority statuses, nor between country and self-perceived minority statuses.

**Figure 2.**
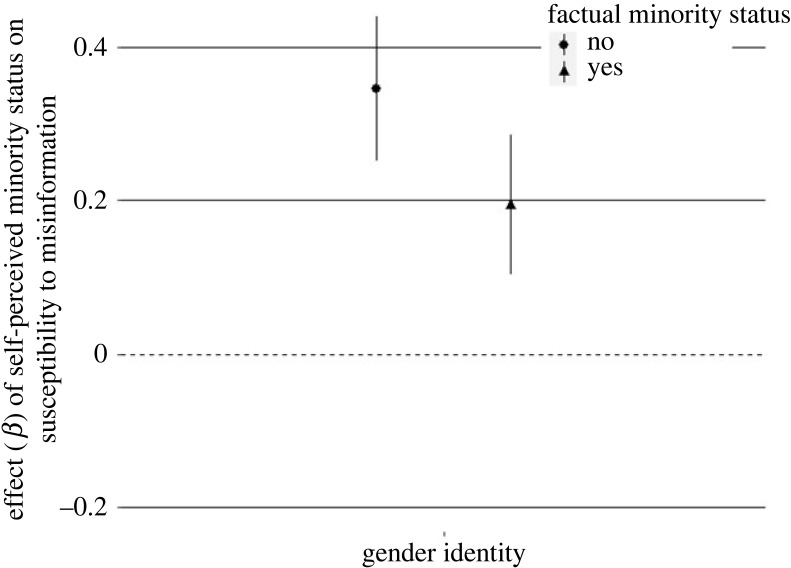
Effect of self-perceived minority status on misinformation susceptibility depending on factual minority status in study 1. Note*:* plots indicate standardized *β* coefficients with 95% confidence intervals.

There was a significant interaction between self-perceived and factual minority statuses based on gender identity. The effect of perceiving oneself as belonging to a minority based on one's gender identity was significantly different depending on one's factual gender group (figure 2). The more participants factually belonging to a gender minority group (i.e. women, non-binary) perceived themselves as part of a minority, the more susceptibility to misinformation they showed, *β* = 0.19, 95% CI [0.10, 0.29], *p* < 0.001.

However, this effect was substantially stronger among participants who factually belonged to the majority group (i.e. men), *β* = 0.35, 95% CI [0.25, 0.44], *p* < 0.001.

#### 3.2.2. Three-way interactions

In the last step of the ANCOVA (table 5, model 3), we added three-way interaction terms (i.e. factual minority status × self-perceived minority status × country for each minority-group type). The analysis revealed no significant three-way interactions.

### 3.3. Discussion

The present research suggests that the perception of belonging to a minority group, rather than factually belonging to it, is most consistently associated with an increased susceptibility to COVID-19 misinformation. Indeed, factually belonging to a minority group was in several cases related to less susceptibility to misinformation. The results highlight that perceiving that one belongs to a minority group may be a primary risk factor for susceptibility to misinformation, at least for some minority dimensions. Perceiving oneself to be a minority in terms of gender identity, ethnicity or region of residence was often related to a higher susceptibility to misinformation. In the case of gender identity, these effects were particularly present when participants did not factually belong to a minority group. However, given the correlational design of our study we cannot establish the causal mechanisms between the variables of interest. It is possible that perceived minority status leads to more misinformation, but the opposite is also possible. For instance, individuals who are susceptible to misinformation may be more likely to consume information that falsely presents them as a minority group. Furthermore, the exact underlying processes giving rise to the observed effects in our work remain unclear, warranting further investigation into the causal mechanisms and potential mediators (e.g. relative deprivation, status threat) that may explain this effect. Moreover, we did not differentiate between perceptions of belonging to a *numerical* minority or a *low-power* minority group in the current study. In some cases (e.g. for male participants), minority perceptions may have primarily been based on beliefs that one belongs to a powerless or marginalized group rather than being the numerical minority. The next study aimed to address these issues.

## 4. Study 2

In study 2, we sought to expand on our findings from study 1 by experimentally testing whether self-perceived minority status is causally related to misinformation susceptibility. Given that we observed the strongest association between self-perceived minority status based on gender identity in the USA, and specifically among men, we created a short experimental video with the aim of testing whether priming adult men from the USA with the perception that men are becoming a minority in important societal domains (i.e. in higher education and the workforce) will be causally related to increased beliefs in misinformation. As COVID-19 had become a far less salient topic in public discourse when we conducted this study than when we conducted the previous study (also see [51]), we used a different outcome measure than susceptibility to COVID-19 misinformation. Specifically, we opted to use a measure of gender conspiracy beliefs [52], which are topically related to our experimental manipulation. We also included the conspiracy mentality questionnaire [53] as a more general measure of conspiracy beliefs.

We tested the following predictions. First, the participants in the experimental condition were expected to display higher levels of conspiracy mentality and gender conspiracy beliefs compared to those in the control condition. Second, this effect was expected to be particularly prominent among individuals who identify as politically right-wing or Republican and those with high SDO, as these ideological factors have been linked with conspiracy beliefs in previous work (e.g. [54,55]). Third, as several studies have highlighted the role of perceived ingroup disadvantage and deprivation to conspiracy beliefs (e.g. [38,40,42,56]), we expected increased levels of collective narcissism, system identity threat and/or group relative deprivation elicited by our minority manipulation to mediate the relationship between the experimental condition and conspiracy mentality/gender conspiracy beliefs. Although collective narcissism is typically seen as a belief about one's ingroups exceptionality, it should be underscored that this belief is thought to be rooted in perceptions of underappreciation and disadvantage [39], which may be nurtured by minority perceptions. Additionally, as research has suggested that perceived threat leads to a more rigid cognition [57], we expected that decreased AOT would mediate the relationship, a construct that has shown to be a robust predictor of misinformation susceptibility [41]. In the absence of experimental effects, we pre-registered to analyse the data correlationally, treating the two manipulation checks (i.e. perceived numerical and perceived low-power minority status) as theoretical predictors, which would provide nuanced insights into the role of different facets of minority perceptions.

### 4.1. Method

#### 4.1.1. Participants

Given our moderation analyses we had to collect a minimum of 1820 participants to obtain 80% power to detect a medium effect size (*d* = 0.50) with 50% attenuation in one condition (see [58]). From 3 May to 2 June 2023, we recruited a sample of adult men from the USA through the online survey platform Prolific. As pre-registered, a total of 141 participants were excluded from the final analysis as they either failed two attention checks (*n* = 3), indicated another gender than male (*n* = 48), did not view the full video (*n* = 45) or had technical issues with audio and/or video (*n* = 45). As these were replaced with new participants in line with Prolific's standards, the final sample consisted of *n* = 1823 men between the ages of 18 and 86 (*M*_age_ = 39.47, s.d._age_ = 13.13). The sample was recruited to accurately mirror the most current distribution of political party affiliation in the USA at the time of data collection (Republican: *n* = 530, Democrat: *n* = 504, Independent: *n* = 745, other/no preference: *n* = 44; [59]). More detailed descriptive statistics can be found in the electronic supplementary material, table S7.

#### 4.1.2. Procedure and materials

This study was approved by the Institutional Review Board of the department of the first author (no. 24949873) and all hypotheses and analyses were pre-registered at https://osf.io/bjgzv?view_only=cc6106c4099a4bbcb3316ed623a4cc87. After consenting to take part in the survey, participants were presented with measures of the moderator variables (i.e. SDO, political orientation), followed by the experimental procedure, the main questionnaire, demographics and a debrief explaining the purpose of the current project. All participants were financially compensated for their participation (equivalent to £9 h^−1^). Exploratory factor analyses with maximum-likelihood estimation were conducted to test the structure of our measures. For all measures we obtained one-factor solutions except for group relative deprivation. Here, one item had to be excluded to obtain a unifactorial solution. Hence, all instruments and items presented below are based on one-factor solutions. All scales were computed by mean-averaging the items belonging to their respective instruments. The R code, and dataset can be found in the electronic supplementary material via OSF: https://osf.io/65jrv/?view_only=4cc3c300fe77470b86cc20f1c098e1ae.

*Moderators.* Participants responded to the SDO measure [37] which contains eight items (e.g. ‘some groups of people are simply inferior to other groups'; *α* = 0.91) that are rated from 1 (*strongly oppose*) to 7 (*strongly favour*). Next, they indicated their political orientation from 0 (*very left-wing/liberal*) to 10 (*very right-wing/conservative*), and whether they generally consider themselves as either a Republican, Democrat, Independent or something else. After responding to these moderator variables, the participants were randomly allocated to either an experimental condition or a control condition as described in the next section.

*Experimental procedure.* In the experimental condition, the participants first viewed a short (approx. 2.5 min.) video, which explained that men have become a minority in higher education and at college campuses, as well as how men are falling behind and struggling in the USA workforce. Next, they were prompted to write briefly about any other societal domains in which men are, or might become, a minority. Participants in the control condition viewed a neutral video, which provided general information about higher education and the workforce, and were prompted to write briefly about it. Both videos were matched in terms of total length and relative proportion of the higher education and workforce content. For the videos, please see the electronic supplementary materials. Having watched the video, participants completed two manipulation checks which asked to what extent they agreed or disagreed that men are becoming a *numerical* minority in society, and a minority in terms of *power and influence* in society. Both questions were rated from 1 (*strongly disagree*) to 7 (*strongly agree*) and were moderately correlated (*r* = 0.57, *p* < 0.001).

*Main questionnaire.* Next, they completed six measures in randomized order. Unless stated otherwise, all measures were rated from 1 (*strongly disagree*) to 7 (*strongly agree*). The conspiracy mentality questionnaire [53] was measured on a scale from 0% (*certainly not*) to 100% (*certainly*) with five items (e.g. ‘I think that there are secret organizations that greatly influence political decisions'; *α* = 0.89). Belief in gender conspiracy beliefs [52] was measured from 1 (*completely disagree*) to 7 (*completely agree*) with three items (e.g. ‘gender ideology was created in order to destroy the Christian tradition’; *α* = 0.89). System identity threat [42] was measured from 1 (*strongly disagree*) to 6 (*strongly agree*) with six items (e.g. ‘there are a growing number of people in this country who have no idea what it means to truly be an American’; *α* = 0.95). Collective narcissism [43] consisted of five items specifically framed for men (e.g. ‘if men had a major say in the world, the world would be a much better place’; *α* = 0.88). Group relative deprivation [19] consisted of five items also specifically framed for men (e.g. ‘I feel furious about men's limited opportunities to get ahead in our lives'; *α* = 0.93). AOT [44] was measured with eight items (e.g. ‘people should take into consideration evidence that goes against their beliefs’; *α* = 0.83). Finally, participants responded to standard demographic measures of age, self-described gender, level of education, employment status, race and whether they were able to view the experimental video without video/audio issues.

#### 4.1.3. Analyses

The analyses were conducted in IBM SPSS v.29 and R v.4.0.3. Mediation analyses were conducted with the PROCESS macro in R [60]. We used the jtools packages [61] to summarize regression results, and the boot packages [62] to bootstrap CIs of differences between regression coefficients. Independent *t*-test was conducted to examine differences between conditions on the dependent variables, mediators and manipulation checks. Next, a two-step multiple regression was conducted twice—once for each dependent variable (i.e. gender conspiracy beliefs and conspiracy mentality). In the first step, we entered condition and the main effects of the moderating variables (i.e. SDO and political orientation), before we in step two entered the interaction terms between condition and SDO, and between condition and political orientation. Finally, we tested two parallel mediation models to examine whether the effects of condition on gender conspiracy beliefs and conspiracy mentality were mediated by system identity threat, collective narcissism, group relative deprivation and AOT. As pre-registered, we tested correlational models with the manipulation checks as independent variables if no experimental effects were found.

### 4.2. Results

First, we tested for multicollinearity among the main variables. Table 6 presents the means, s.d. and correlations between all main study variables. No correlations suggested that multicollinearity would be an issue, and further testing with collinearity statistics in a regression analysis confirmed this (i.e. lowest tolerance = 0.36; highest variance inflation factor = 2.79; e.g. [63,64]).

**Table 6.** Correlations, means and s.d. for study variables in study 2. (SDO, social dominance orientation; SIT, system identity threat; CNS, collective narcissism; GRD, group relative deprivation; AOT, actively open-minded thinking; GCB, gender conspiracy beliefs; CMQ, conspiracy mentality questionnaire.)

variables		*M*	s.d.	1	2	3	4	5	6	7	8	9
1.	numerical minority	3.56	1.63	—								
2.	power minority	3.51	1.65	0.57*	—							
3.	SDO	2.67	1.41	0.20*	0.30*	—						
4.	political orientation^a^	5.66	2.82	0.24*	0.30*	0.55*	—					
5.	SIT	4.08	1.76	0.34*	0.40*	0.48*	0.53*	—				
6.	CNS	2.85	1.36	0.39*	0.46*	0.45*	0.42*	0.55*	—			
7.	GRD	2.59	1.45	0.43*	0.55*	0.44*	0.37*	0.54*	0.75*	—		
8.	AOT	5.33	0.91	−0.25*	−0.28*	−0.42*	−0.35*	−0.38*	−0.44*	−0.44*	—	
9.	GCB	3.11	1.15	0.29*	0.37*	0.51*	0.54*	0.64*	0.55*	0.54*	−0.38*	—
10.	CMQ	7.05	2.30	0.19*	0.21*	0.22*	0.22*	0.44*	0.32*	0.32*	−0.22*	0.38*

^a^Higher values indicate a more right-leaning political orientation.

*p < 0.001.

Next, we ran a series of one-tailed independent *t*-tests to see whether the experimental condition significantly differed from the control condition on the dependent variables, mediators and manipulation checks (table 7). In terms of the manipulation checks, the results indicated that, when compared to the control condition, participants in the experimental condition agreed significantly stronger that men are becoming a numerical minority and a minority in terms of power and influence in society, but these effects were small. We also observed a very small significant difference in conspiracy mentality where the control condition against expectations scored higher than the experimental condition. Following up on this unexpected finding, Bayesian statistics showed that there was moderate evidence favouring no effect of condition on conspiracy mentality (Bayes factor_01_ = 6.09). No other significant differences were observed.

**Table 7.** Independent *t*-tests between conditions on dependent variables, mediators and manipulation checks in study 2. (SIT, system identity threat; CNS, collective narcissism; GRD, group relative deprivation; AOT, actively open-minded thinking; GCB, gender conspiracy beliefs; CMQ, conspiracy mentality questionnaire.)

variables	experiment (*n* = 884)		control (*n* = 939)		*t*	*p*-value	Cohen's *d*
	*M*	s.d.	*M*	s.d.			
numerical minority	3.87	1.61	3.28	1.59	7.86	<0.001	0.37
power minority	3.76	1.66	3.28	1.61	6.25	<0.001	0.29
SIT	4.07	1.75	4.08	1.77	0.11	0.456	0.01
CNS	2.85	1.38	2.85	1.34	0.06	0.478	0.00
GRD	2.63	1.46	2.56	1.44	0.99	0.160	0.05
AOT	5.35	0.90	5.31	0.92	0.62	0.266	0.03
GCB	3.11	1.14	3.11	1.16	0.21	0.417	0.01
CMQ	6.96	2.32	7.14	2.28	1.69	0.045	0.08

Following these group comparisons, we tested whether the effect of condition on gender conspiracy beliefs was moderated by SDO and political orientation. A two-step multiple regression analysis was conducted where we in the first step entered condition and the two moderators, whereas the interaction terms between condition and the moderators were entered in the second step. The regression results are presented in table 8. The results indicated that gender conspiracy beliefs were significantly predicted by SDO, and political orientation, but no significant interaction between condition and the moderators emerged. Please note that the interaction was also non-significant when political orientation was replaced with the categorical political affiliation measure.

**Table 8.** Stepwise multiple regression results for gender conspiracy beliefs and conspiracy mentality. *n* = 1823. GCB, gender conspiracy beliefs; CMQ, conspiracy mentality questionnaire; SDO, social dominance orientation. In step 1, we examined the impact of condition (control versus experimental), SDO and political orientation on GCB and CMQ. In step 2, we entered the interaction terms between condition and SDO, and between condition and political orientation. *p_h_* = Holm-corrected significance level.)

variables	model 1: GCB				model 2: CMQ			
	*B*	*β*	s.e. *B*	*p_h_*	*B*	*β*	s.e. *B*	*p_h_*
step 1								
constant	3.10	3.10	0.03	<0.001	7.13	7.13	0.07	<0.001
condition^a^	0.02	0.02	0.04	0.559	−0.15	−0.15	0.10	0.135
SDO	0.25	0.43	0.02	<0.001	0.23	0.32	0.04	<0.001
political orientation^b^	0.15	0.35	0.01	<0.001	0.11	0.33	0.02	<0.001
*R*^2^	0.35				0.06			
step 2								
constant	3.10	3.10	0.03	<0.001	7.13	7.13	0.07	<0.001
condition^a^	0.02	0.02	0.04	0.563	−0.15	−0.15	0.10	0.410
SDO	0.29	0.39	0.03	<0.001	0.20	0.28	0.06	0.006
political orientation^b^	0.14	0.39	0.01	<0.001	0.10	0.28	0.03	0.006
condition × SDO	−0.07	−0.10	0.04	0.224	0.07	0.09	0.09	0.946
condition × political orientation	0.03	0.08	0.02	0.173	0.03	0.09	0.04	0.946
*R*^2^	0.36				0.06			
Δ*R*^2^	0.01				0.00			

^a^Control condition = 0, experimental condition = 1.

^b^Higher values indicate a more right-leaning political orientation.

The same procedure was followed once more with conspiracy mentality as the dependent variable. Here, both SDO and political orientation were significantly associated with conspiracy mentality, but again, no significant interaction effect was found between condition and the moderators. Again, the interaction was also non-significant when political orientation was replaced with the categorical political affiliation measure. Owing to the lack of experimental effects of condition on the mediators, we did not test for mediation.

#### 4.2.1. Perceived minority status as independent variable

Given that the results we obtained did not support our main predictions, as pre-registered, we repeated the same moderation and mediation analyses once more with the two manipulation checks as independent variables instead of condition. Specifically, we conducted a stepwise multiple regression analysis to first test whether perceiving men as a numerical minority in society, and perceiving men as a minority in terms of power and influence in society, were related to increased gender conspiracy beliefs and an increased conspiracy mentality. In step 2, we tested whether these effects were moderated by SDO and political orientation. The results from the two stepwise regression models are displayed in table 9.

**Table 9.** Stepwise multiple regression results for gender conspiracy beliefs and conspiracy mentality with manipulation checks as independent variables. (*n* = 1823. GCB, gender conspiracy beliefs; CMQ, conspiracy mentality questionnaire; SDO, social dominance orientation. In step 1, we examined the impact of perceptions of men being a numerical minority, perceptions of men being a minority in terms of power and influence, SDO and political orientation on GCB and CMQ. In step 2, we entered the interaction terms between minority perceptions and SDO, and between minority perceptions and political orientation. *p_h_* = Holm-corrected significance level.)

variable	model 1: GCB				model 2: CMQ			
	*B*	*β*	s.e. *B*	*p_h_*	*B*	*β*	s.e. *B*	*p_h_*
step 1								
constant	2.56	3.11	0.06	<0.001	6.15	7.05	0.14	<0.001
numerical minority	0.05	0.08	0.02	0.002	0.12	0.20	0.04	0.001
power minority	0.11	0.18	0.02	<0.001	0.13	0.22	0.04	<0.001
SDO	0.21	0.30	0.02	<0.001	0.18	0.26	0.04	<0.001
political orientation^a^	0.14	0.38	0.01	<0.001	0.09	0.25	0.02	<0.001
*R*^2^	0.39				0.08			
step 2								
constant	2.56	3.11	0.06	<0.001	6.16	7.06	0.14	<0.001
numerical minority	0.05	0.08	0.02	0.009	0.12	0.20	0.04	0.011
power minority	0.11	0.18	0.02	<0.001	0.13	0.22	0.04	0.007
SDO	0.18	0.29	0.05	<0.001	0.21	0.26	0.12	0.439
political orientation^a^	0.17	0.39	0.02	<0.001	0.09	0.25	0.06	0.549
numerical minority × SDO	−0.01	−0.02	0.01	>0.999	0.02	0.05	0.03	>0.999
numerical minority × political orientation	−0.01	−0.03	0.01	>0.999	−0.00	−0.02	0.02	>0.999
power minority × SDO	0.01	0.03	0.01	>0.999	−0.03	−0.06	0.03	>0.999
power minority × political orientation	0.00	−0.01	0.01	>0.999	0.00	0.02	0.02	>0.999
*R*^2^	0.39				0.09			
Δ*R*^2^	0.00				0.01			

^a^Higher values indicate a more right-leaning political orientation.

In the first step of the regression analysis, results showed that perceptions of men becoming a numerical minority in society, perceptions of men becoming a minority in terms of power and influence in society, SDO and political orientation all were significantly and positively related to gender conspiracy beliefs and conspiracy mentality. In step 2, we found no significant interactions between our independent variables and moderators on neither gender conspiracy beliefs nor conspiracy mentality. These results suggest that the association of perceiving men as a numerical minority and a minority in terms of power and influence with both gender conspiracy beliefs and conspiracy mentality does not statistically significantly depend on the level of SDO or political orientation.

Additionally, we also tested in step 1 whether the regression coefficients of the two manipulation checks on gender conspiracy beliefs and conspiracy mentality were significantly different from each other. Here, we calculated 95% CIs for the difference with 5000 bootstrap resamples of our data. The results showed that perceptions of men as a low-power minority had a significantly stronger association with gender conspiracy beliefs than perceptions of men as a numerical minority, Δ*B* = 0.06, 95% CI [0.01, 0.11], whereas no significant difference was found between the two minority perceptions on conspiracy mentality, Δ*B* = 0.01, 95% CI [−0.12, 0.14].

*Correlational mediation models.* Parallel mediation models were set up to test whether the associations between the manipulation checks (i.e. perceiving men as a numerical minority, and perceiving men as a minority in terms of power and influence) with gender conspiracy beliefs and conspiracy mentality were mediated by system identity threat, collective narcissism, group relative deprivation and AOT. We entered the latter variables simultaneously as four parallel mediators in every model. In the first model we entered gender conspiracy beliefs as the dependent variable, whereas conspiracy mentality was entered as the dependent variable in the second model. Both manipulation checks were entered simultaneously as predictors. For parsimony, we visualize the effects of each predictor separately, but it is important that these effects are based on the same model and control for the effects of the respective other minority perception item.

**Figure 3.**
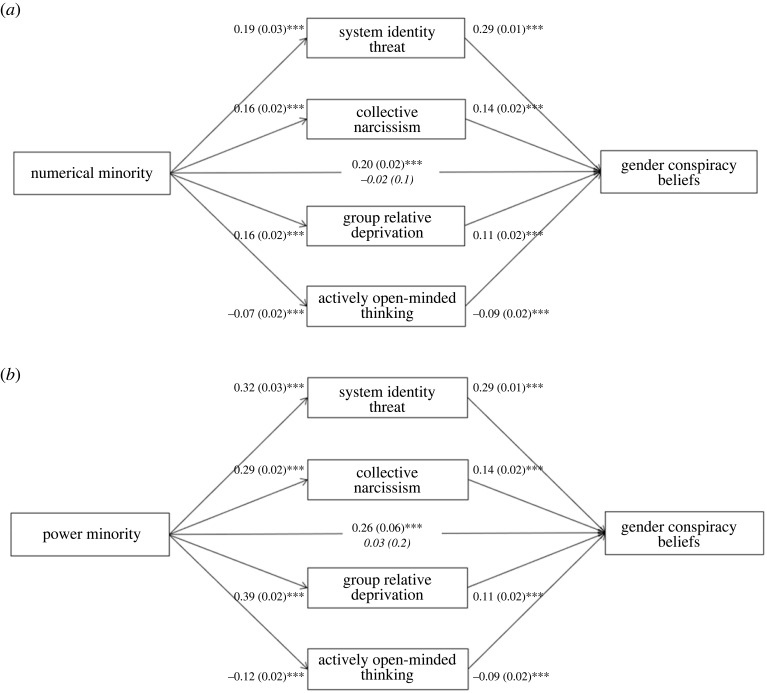
Parallel mediation models with perceived numerical minority status (*a*), perceived power minority status (*b*), and gender conspiracy beliefs in study 2. Note: coefficients are *B* (s.e.). Adjusted direct effects are in italics. The estimates are based on the same model in which both minority perception variables are entered simultaneously. ***p < 0.001.

First, we tested a model with gender conspiracy beliefs as the dependent variable. Both perceived numerical minority status and perceived minority status in terms of power and influence were positively associated with system identity threat, collective narcissism, group relative deprivation and negatively associated with AOT. Each of these mediators was positively related to gender conspiracy beliefs. The results with perceived numerical minority status as the independent variable are displayed in figure 3*a*. The indirect effects of perceived numerical minority status through system identity threat, *β* = 0.08, s.e. = 0.01, 95% CI [0.05, 0.11], collective narcissism, *β* = 0.03, s.e. = 0.01, 95% CI [0.02, 0.05], group relative deprivation, *β* = 0.02, s.e. = 0.01, 95% CI [0.01, 0.04] and AOT, *β* = 0.01, s.e. = 0.01, 95% CI [0.00, 0.02], were significant.

The association for perceptions of men as being a minority in terms of power and influence as independent variable are shown in figure 3*b*. The indirect effect through system identity threat, *β* = 0.13, s.e. = 0.01, 95% CI [0.11, 0.16], collective narcissism, *β* = 0.06, s.e. = 0.01, 95% CI [0.04, 0.08], group relative deprivation, *β* = 0.06, s.e. = 0.02, 95% CI [0.03, 0.09] and AOT, *β* = 0.02, s.e. = 0.01, 95% CI [0.01, 0.03], were all significant. Taken together, these findings indicate that perceiving men as both a numerical minority and as a low-power minority are associated with higher gender conspiracy beliefs owing to higher system identity threat, collective narcissism, group relative deprivation and less AOT.

**Figure 4.**
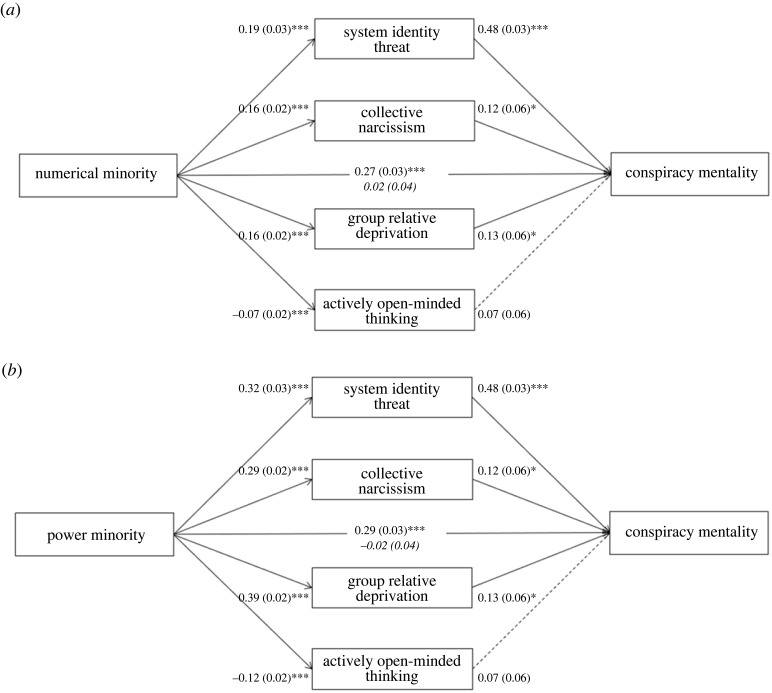
Parallel mediation model with perceived numerical minority status (*a*), perceived power minority status (*b*), and conspiracy mentality. Note: coefficients are *B* (s.e.). Adjusted direct effects are in italics. The estimates are based on the same model in which both minority perception variables are entered simultaneously. *p < 0.01, ***p < 0.001. Dotted lines are non-significant (*p* = 0.191).

We tested the mediation model again, this time with conspiracy mentality as the dependent variable. Of the mediators, system identity threat was moderately and collective narcissism and group relative deprivation weakly positively related to conspiracy mentality. The association between AOT and this dependent variable was not statistically significant. The results with perceived numerical minority status as the independent variable are displayed in figure 4*a*. The indirect effects through system identity threat, *β* = 0.06, s.e. = 0.01, 95% CI [0.04, 0.09], and group relative deprivation, *β* = 0.02, s.e. = 0.01, 95% CI [0.00, 0.03], were significant. The indirect effects through collective narcissism, *β* = 0.01, s.e. = 0.01, 95% CI [−0.00, 0.03], and AOT, *β* = −0.00, s.e. = 0.00, 95% CI [−0.01, 0.00], were non-significant. These findings suggest that perceiving men to be a numerical minority in society is related to higher conspiracy mentality owing to higher system identity threat and group relative deprivation, rather than collective narcissism and AOT.

The effects of perceived minority status in terms of power and influence are presented in figure 4*b*. The indirect effects through system identity threat, *β* = 0.11, s.e. = 0.01, 95% CI [0.08, 0.14], collective narcissism, *β* = 0.02, s.e. = 0.01, 95% CI [0.00, 0.05] and group relative deprivation, *β* = 0.04, s.e. = 0.02, 95% CI [0.01, 0.07], were significant. The indirect effect through AOT, *β* = −0.01, s.e. = 0.01, 95% CI [−0.02, 0.00], was non-significant. These results indicate that perceiving men to be a minority in terms of power and influence is related to higher conspiracy mentality owing to higher system identity threat, collective narcissism and group relative deprivation.

Notably, the indirect effects in both models were generally stronger for the power minority perceptions, owing to significantly stronger associations between the power minority perception (as compared to numerical minority perception) with system identity threat, Δ*B* = 0.13, 95% CI [0.02, 0.24], collective narcissism, Δ*B* = 0.13, 95% CI [0.06, 0.21] and group relative deprivation, Δ*B* = 0.23, 95% CI [0.15, 0.31]. There was no significant difference between the associations with AOT, Δ*B* = −0.04, 95% CI [−0.09, 0.01].

### 4.3. Discussion

We found no support for our main predictions in study 2 as no significant predicted experimental effects were observed on gender conspiracy beliefs nor on conspiracy mentality and one effect went in the opposite direction. Furthermore, against expectations, these effects were not significantly moderated by SDO or political orientation. The weak effect on the manipulation checks may suggest that our manipulation was too weak to produce downstream effects on the outcome measures. Alternatively, it is possible that minority perceptions are not causally related to conspiracy beliefs. We return to a discussion of these possibilities and the unexpected effect in the general discussion.

Nevertheless, the pre-registered correlational results from study 2 largely corroborated the findings from study 1, showing that men who perceive themselves as a minority based on their gender are more susceptible to misinformation in the form of gender conspiracy beliefs and they also exhibit higher levels of a general conspiracy mentality. With the manipulation checks as independent variables, we found that both perceptions of being a numerical minority and a power minority were associated with stronger gender conspiracy beliefs and a higher conspiracy mentality, although the associations with gender conspiracy beliefs was significantly stronger for perceiving men as a minority in terms of power and influence. Moreover, we found that perceptions of being a numerical minority and a minority in terms of power and influence both were associated with having stronger gender conspiracy beliefs through stronger system identity threat, collective narcissism and group relative deprivation. Our results also indicate that lower AOT correlationally mediated the relationships between the two different minority perceptions and gender conspiracy beliefs, but not for conspiracy mentality. However, the indirect effects tended to be stronger for perceptions of being a minority in terms of power. Indeed, the associations between the mediators system identity threat, collective narcissism and group relative deprivation and the power minority perception were significantly stronger than with the numerical minority perception. This finding was important as it suggests that the findings in study 1 may be primarily driven by the perception of being a low-power minority group. Nevertheless, when interpreting these findings, it is essential to keep in mind that they are based on correlations that provide little evidence for the causal direction assumed in the models [65,66].

## 5. General discussion

We demonstrated that having a self-perceived minority status (studies 1 and 2), rather than factually being part of a minority group (study 1), is most consistently related to misinformation susceptibility. Across national samples, factually belonging to a minority group was in several cases related to lower misinformation susceptibility in the first study. Conversely, perceiving oneself as a minority in terms of gender identity, ethnicity or region of residence was often related to a higher susceptibility to misinformation. For gender identity, this association was particularly present when people did not factually belong to the minority group (i.e. the effect was strongest for men). In the second study, we failed to find experimental effects but correlationally identified system identity threat, collective narcissism, group relative deprivation and to some extent, AOT as potential processes behind the association between perceiving oneself as a gender minority and misinformation susceptibility in the form of gender conspiracy beliefs and conspiracy mentality. Moreover, we showed that perceptions of being a low-power minority generally showed stronger relationships with the mediators and one of the dependent variables than perceptions of being a numerical minority.

The present research extends work showing that perceived ingroup disadvantage is linked to susceptibility to misinformation in the context of conspiracy beliefs (e.g. [38,40]). Existing perspectives view conspiracy beliefs as motivated reactions to perceived threats (e.g. [67,68]), arguably in an attempt to reduce the accompanying negative states such as anxiety and uncertainty. For instance, it is likely that some men feel threatened and see themselves as a minority in today's society because they perceive male gender norms as being challenged by societal shifts (see [69]). In line with previous work [42], we demonstrate that this kind of ‘system identity threat’ or the perception that fundamental societal values and norms are under attack are indeed underlying the positive correlation between minority perceptions and conspiracy belief.

Corresponding with previous work, we also observed significant indirect associations through collective narcissism and group relative deprivation. Across multiple countries, both belief and willingness to spread conspiracy theories have been associated with collective narcissism [40], a belief that one's ingroup (in our study, men) is exceptional yet underappreciated, and rooted in perceptions of being disadvantaged [39]. Relative deprivation has also been found to be associated with increased conspiracy beliefs in Poland [38] and the Netherlands [14]. Taken together, although no experimental effects were observed, our results tentatively support the notion that perceived ingroup disadvantage may be a driver of conspiracy beliefs whether it is in terms of system identity threat, collective narcissism or relative deprivation.

Recent work has showed that an AOT style [70] is a notably strong negative predictor of susceptibility to misinformation [41]. In line with this, we find that the effect of both types of minority perceptions on gender conspiracy beliefs are to some extent driven by lower AOT. In line with recent research showing that threat perceptions elicit rigid cognitive styles [57], minority perceptions may reduce AOT. Participants who believe in gender conspiracy theories may, therefore, be individuals who in response to status threats hold particularly strong convictions on the topic of gender and are generally less willing to change their views in light of contradicting information. However, there was no significant indirect effect through AOT on conspiracy mentality. This finding was surprising but may suggest that AOT is primarily linked to the belief in specific conspiracy theories (i.e. regarding gender) rather than general conspiracy tendencies.

In study 2, perceiving men as a numerical minority and as a minority in terms of power and influence both were significantly related to higher conspiracy beliefs. There were nevertheless some notable differences. Generally, the perception of being a minority in terms of power and influence was more strongly related to most mediators and at least one of the dependent outcomes, thereby producing stronger indirect effects. These differences suggest that especially a lack of influence rather than simply being numerically under-represented in certain societal contexts underlies the link with conspiracy beliefs. Nevertheless, we must emphasize that our reliance on single items inhibited our ability to ascertain whether participants made clear distinctions between the two types of minority perceptions. The moderate correlation observed between these items suggests some shared conceptual core, yet it also implies that they might capture distinct facets of the overarching phenomenon. Therefore, future research should employ multi-item measures when examining minority perceptions to delineate more accurately the boundaries of the constructs. Furthermore, subsequent studies should consider formulating questions that are directly tailored to specific domains, such as education or the workforce, rather than society generally. When questions are posed considering society generally, there is a possibility that participants may interpret them in the context of power and influence, as opposed to strictly numerical representation. This is especially pertinent given the implausibility of the notion that men, as a demographic, are diminishing in overall societal numbers.

In our two studies we employed different measures of our dependent variables. In study 1, we included a measure of susceptibility to COVID-19 misinformation given the high salience of the topic at that time. In study 2, we used a measure of gender conspiracy beliefs and the conspiracy mentality questionnaire. Although this limits the comparability between studies, we correlationally find evidence that self-perceived minority status based on gender is robustly associated with the susceptibility to different kinds of misinformation and conspiracy beliefs. Importantly, our results underscore that perceiving oneself as being a gender minority, even though one factually belongs to the majority group, can be a primary risk factor for endorsing misperceptions on a variety of topics. Future research is needed to test whether this assumption also extends to misinformation on other topics (e.g. climate change) and other specific conspiracy theories.

Across countries (study 1), we observed that belonging to the far left of the political spectrum or being non-religious/atheist was associated with lower susceptibility to COVID-19 misinformation. By contrast, belonging to the far right of the political spectrum was associated with more susceptibility. Previous research has suggested that, although people on either extreme end of the political spectrum score higher on conspiracy mentality, this relationship is skewed towards the extreme right [55], consistent with our results from study 2 and other recent work [71]. Here, we further show that specific misbeliefs about COVID-19 may be more prevalent among the far-right minority. However, it could be argued that another selection of misbeliefs could have produced higher scores among left-wing individuals. Indeed, all the items were positively correlated with a right-wing political orientation. At the same time, because of strong political polarization on COVID-19 [72], right-leaning media has more frequently propagated inaccurate claims about COVID-19 [73], and this exposure may explain the current findings. We also found significant associations between having a right-wing political orientation and increased gender conspiracy beliefs in study 2. These associations may reflect that individuals on the political right, as argued by Graff *et al.* [74], are greatly committed to gender conservatism, an essentialized understanding of gender, and traditional family values.

Replicating and nuancing previous research [13,75], we find that belonging to a religious group is related to higher susceptibility to COVID-19 misinformation regardless of whether this group is the societal majority or minority group. Compared to non-believers, religious people have been found to require less evidence before believing new claims that are presented to them [76]. This tendency might explain why those belonging to religious groups in the present research showed a higher susceptibility to misinformation. Importantly, religious majority and minority group members did not differ in their beliefs in misinformation. Thus, attempts to counter such misbeliefs may focus on believers generally or the religious majority to avoid the further stigmatization of often marginalized religious minority groups.

In terms of region of residence, we also find that minority members (i.e. rural residents) show less susceptibility to COVID-19 misinformation. To some extent, this contrasts with previous cross-cultural research which found that urban residents across 16 countries reliably showed lower susceptibility to misinformation [77]. However, it is important to note that the category ‘urban’ encompasses many different types of neighbourhoods, some of which may be privileged whereas others may be disadvantaged. As such, some urban participants may reside in underprivileged neighbourhoods and experience feelings of relative deprivation—feelings which previously have been linked to increased conspiracy beliefs [14,38]. As such, more refined measures may have produced different results.

Although some nuances and exceptions were observed, our results overall emphasize that the perception of belonging to a minority group, rather than factually belonging to one, may be the most consistent risk factor for or at least a robust indicator of misinformation susceptibility. This entails some practical implications for researchers and policymakers. As believing and acting upon misperceptions about pandemics or other health crises may have serious health consequences [78,79], it is of utmost importance to develop strategies and interventions to combat it [80]. Researchers and policymakers should devote resources and attention to reaching those who perceive themselves as belonging to a minority group or otherwise feel marginalized and powerless, potentially via local community leaders [81]. However, future research is needed to establish whether reducing people's minority perception is a fruitful way to reduce beliefs in misinformation or whether different causal pathways are at play.

Our first study cannot establish the causal mechanisms between the variables of interest and the second study failed to find consistent experimental effects. The only effect observed was the opposite of our predictions, with scores on the conspiracy mentality questionnaire being slightly *lower* in the experimental condition than in the control condition. However, as indicated by Bayesian statistics, this unexpected effect may have been a possible false positive. Indeed, we employed one-tailed significance tests based on our unidirectional hypotheses. Using a more stringent two-tailed test would have rendered the difference non-significant. Thus, we caution against interpreting this effect as meaningful until it is confirmed in subsequent research.

Several reasons could account for the null findings in study 2. For instance, the manipulation may not have been potent enough to have downstream consequences on conspiracy beliefs. Relatedly, the experiment may have been underpowered if the effect was much lower than we anticipated. Support for both the former and latter possibility is provided by the relatively small effect on the manipulation checks, which typically is larger than effects on dependent variables. It is also possible that perceived minority status, as observed among majority group members like men in study 2, can be contextualized within a broader network of conspiracy beliefs. That is, minority status perceptions could be viewed as one specific conspiracy belief, which typically tends to be positively related with other conspiracy beliefs [82]. As such, these theories may be correlated but not causally interconnected.

Another conceivable explanation may be that there is no causal link between perceiving oneself as a minority member and believing in misinformation and conspiracy theories and that both are caused by a third variable. Perceiving oneself as a minority might be influenced by several contextual parameters which may also be causally related to misinformation susceptibility. For instance, people who perceive themselves to be marginalized and deprived may do so because they perceive the political and social system as rigged (e.g. [14]). This tendency may also lead them to rely more on alternative sources of information rather than official accounts. Alternatively, it is also possible that the sources of misinformation and conspiracy theories also spread false information that suggests their consumers are a minority group in society. This could especially be the case in terms of sources that spread gender conspiracy theories, where traditional gender roles and norms are viewed as threatened by progressive gender policies. Moreover, conspiracy theories often suggest that powerful groups control ordinary people (e.g. [50,83]), which may contribute to individuals' perception of belonging to a minority group. Hence, the observed results from study 2, which did not identify a direct causal influence of minority perceptions on conspiracy mentality, may have potentially been influenced by unobserved third variables. For instance, both a general conspiracy mentality and minority perceptions can emerge from a perceived threat to the ingroup's status quo. In this sense, such a third variable may have been driving the observed correlations in this research. Alternatively, the reversed implied causation is also possible. Conspiracy mentality in study 2 might be a foundational tendency influencing specific beliefs, such as the notion that men have become a minority. Future work is needed to establish the direction of the proposed processes using experimental and longitudinal designs to uncover causal mechanisms related to minority perceptions and misinformation susceptibility.

The way we conceptualized (in studies 1 and 2) and manipulated (in study 2) minority perceptions in the present research may be ecologically valid given the use of the term in public discourse [84] and distinct from related constructs that commonly involve a negative affective response (e.g. relative deprivation, collective victimhood). The correlational mediation models further suggested that the included constructs are only weakly to moderately related. Nevertheless, we acknowledge the potential conceptual overlap that should be further investigated in future research (see [85]). Furthermore, the mediation analysis indicated that the mediators completely accounted for the potential direct association between minority perceptions and conspiratorial thinking. In other words, the model affirms the convergent validity of our principal construct of interest, given its consistent relation to the mediators. Yet, the models also demonstrate that our primary construct does not have a unique association with conspiracy mentality when these mediators are considered. This finding might challenge the predictive validity of our principal construct.

Lastly, it is important to note that the content of misinformation changes dynamically. Participants in study 1 could have been aware that the specific misinformation we referred to in our items had already been debunked. This may have explained why the susceptibility to misinformation was overall low in our sample. Moreover, as conspiracy theories often are born with major sociopolitical events, the relationships may have shown different patterns in study 2 with a different set of conspiracy beliefs. Although we found relatively consistent relationships between the constructs of interest, future research may investigate whether our findings can be replicated with other types of misinformation.

## 6. Conclusion

The present study suggests that misinformation susceptibility is most consistently associated with having a self-perceived rather than factual minority status. Specifically, men who perceive themselves as a gender minority show higher susceptibility to COVID-19 misinformation, gender conspiracy beliefs and a higher conspiracy mentality. The association with gender conspiracy beliefs and conspiracy mentality are correlationally mediated by perceived ingroup disadvantage in the form of higher system identity threat, collective narcissism, group relative deprivation and lower AOT (only for gender conspiracy beliefs, not for conspiracy mentality). Generally, perceptions of being a minority in terms of power and influence rather than being a numerical minority was most indicative of conspiracy beliefs.

## Data Availability

Dataset, R code and electronic supplementary material analyses: https://osf.io/65jrv/?view_only=4cc3c300fe77470b86cc20f1c098e1ae. The data are provided in the electronic supplementary material [86].

## Footnotes

1 When run without covariate adjustments, the main effect of belonging to a factual minority group based on one's sexual orientation was non-significant (*p* = 0.054), whereas the remaining pattern of results remained the same. See the electronic supplementary material, table S5.
